# Effect of Waste Micro-Particles on Metalworking Fluid Efficiency and Biodegradation During the Cutting Process

**DOI:** 10.3390/ma18173988

**Published:** 2025-08-26

**Authors:** Stepanka Dvorackova, Martin Bilek, Josef Skrivanek, Dora Kroisová, Anita Białkowska, Mohamed Bakar

**Affiliations:** 1 Department of Machining and Assembly, Faculty of Mechanical Engineering, Technical University of Liberec, 46117 Liberec, Czech Republic; stepanka.dvorackova@tul.cz (S.D.); dora.kroisova@tul.cz (D.K.); 2 Department of Textile Machine Design, Faculty of Mechanical Engineering, Technical University of Liberec, 46117 Liberec, Czech Republic; martin.bilek@tul.cz; 3 Department of Machine Design and Materials Science, Faculty of Mechanical Engineering, University of Radom, 26-600 Radom, Poland; a.bialkowska@uthrad.pl

**Keywords:** metalworking fluid (MWFs), cutting, contamination, micro particles, microorganisms, filtration, ozonation, decontamination, *Pseudomonas aeruginosa*

## Abstract

This study investigates contaminants in metalworking fluids (MWFs) from an industrial band saw, focusing on microparticle classification and microbial quantification linked to fluid degradation. Most particles were under 50 µm, primarily aluminum and iron oxides from tool wear; oxygen- and sulfur-containing particles suggested corrosion. Microbiological analysis showed high contamination, with culturable microorganisms exceeding 1000 CFU/mL. A pathogenic strain associated with biodeterioration was identified, underscoring the need for microbial control. Filtration and ozonation have been used as decontamination methods to improve the purity and biological stability of the process fluid. Filtration enabled selective removal of metallic microparticles. Among six nanofiber filters, the Berry filter achieved the highest efficiency (70.8%) for particles ≥ 7.3 µm, while other filters were faster but less efficient. Ozonation proved highly effective for microbiological decontamination, reducing viable microorganisms by over 95%, improving visual clarity, and lowering pH from 9 to 8 while remaining within operational limits. Unlike filtration, ozonation significantly reduced microbial load. The combination of both methods is proposed as a sustainable strategy for maintaining process fluid quality under industrial conditions. These findings support integrated decontamination approaches to extend fluid life, reduce fresh MWF consumption and waste, and enhance workplace hygiene and safety in machining operations.

## 1. Introduction

Metalworking fluids (MWFs) have been known for more than a century as essential components in machining processes such as turning, milling, grinding, drilling, and cutting. They contribute significantly to improving machining efficiency and the quality of treated surfaces [1,2,3,4].

The main functions of MWFs are cooling, lubrication, cleaning, and corrosion protection of the metals and tools. Moreover, proper use of MWFs can improve tool performance, ensure machining quality and accuracy, and increase productivity [5,6,7].

MWFs are more commonly classified as water-based and oil-based fluids, the latter comprising emulsions, semi-synthetic, and synthetic types [1,7,8]. It is important to specify that water-based cutting fluid represents about 90% of all cutting fluids used [9,10]. However, oil-based fluids, in general, contain a base oil (mineral, synthetic, or vegetable), for tribological reasons, and other additives [10,11,12].

A major problem affecting MWFs and particularly those involved in cutting operations, is the contamination by solid particles and microorganisms such as fungi and bacteria which are harmful for the respiratory system and skin of workers [13,14]. Gordon [15] demonstrated that microbial changes that occur in fluid composition, during use and storage in the workplace, are responsible for the respiratory problems of machine operators. However, Rabenstein et al. [16] investigated the microbial degradation of metalworking fluids and discovered that mineral oil-based MWF emulsions were not degraded uniformly as a whole by microorganisms. Indeed, some compounds were subject to rapid microbial degradation, causing adverse effects on health. Similar results regarding the microbial effect on the water-based performance of MWFs have been reported elsewhere [17,18]. In addition, Elansky et al. [19] confirmed the aforementioned changes in the composition of microorganisms during fluid operation and highlighted that microbial degradation remains one of the most efficient ways to dispose of water-miscible MWFs. In a separate work on the effect of solid particles on MWFs, Beekhuis [20] confirmed that contamination by crumbled solid abrasive particles from grinding wheels caused negative effects on the operation of the machine tool.

Manufacturers and researchers have become aware of the growing importance of ecology and have been compelled to explore alternative materials and manufacturing methods to achieve progressive sustainability. In recent years, there have been many efforts to replace mineral oil-based MWFs with environmentally friendly MWFs, while minimizing environmental impact and maintaining the performance required in metalworking processes.

An extensive literature review was conducted on studies related to environmentally friendly metalworking fluids (MWFs) [21,22,23,24,25,26]. Nune and Chaganti [27] prepared an eco-friendly MWF which showed comparable properties with the conventional MWF and was safe for workers. However, Jyothish et al. [28] introduced a base oil and selected additives for developing ecologically benign MWFs, while Sułek and his research group [29] prepared ecological cutting fluids by using surfactants. Due to the improved lubrication, the tested vegetable oils demonstrated remarkable suitability as cutting fluids in industrial applications [30].

Furthermore, it should be emphasized that more efforts are still needed to completely eliminate the use of mineral oil-based metalworking fluids (MWFs). Mineral oil-based metalworking fluids (MWFs) contain toxic and hazardous chemicals, making them dangerous to humans and the environment.

Although MWFs have been used for a very long time, problems regarding their contamination and recycling remain. Various chemical and physical methods have been developed to treat metalworking fluids during and after use. These methods include membrane filtration, coagulation, biodegradation, and disinfection techniques [31,32,33,34,35,36,37,38]. Filtration plays a vital role in removing contaminants and extending the life of lubricants and machine components [31]. Köker and Cebeci used membrane filtration chemical coagulation methods to treat MWFs waste from the metalworking industry [32]. Results showed that the efficiency of waste removal by microfiltration membranes was higher than that of coagulation. This high efficiency of waste removal by membrane filtration is consistent with the results reported in other studies [33,34]. In another study, Saha et al. [35] used a high-intensity irradiation UV lamp for different exposure times under both static and mixed conditions to disinfect metalworking fluids. The obtained results confirmed that successful disinfection of MWFs was reached with mixing combined with high-intensity UV radiation without altering the performance of the fluid activities. The usefulness of UV radiation in the disinfection of MWFs has been confirmed elsewhere [36]. Among other alternative disinfection methods, ozonation seems to be particularly effective in inactivating microorganisms without generating secondary pollutants, thus achieving high sterilization efficiency [37]. Comparative studies have confirmed the strong antimicrobial performance of ozonation [6,38].

Biological treatment of used metal-working fluids appears to be an interesting method because it is efficient with relatively low energy requirements. However, this process is challenging due to the presence of biocides that can delay microbial deterioration during fluid use [39].

In a review study, Milić et al. [40] highlighted the main disadvantages of membranes used in the treatment of spent metalworking fluids and suggested the combination of two or more separation processes to reach optimal process performance. The simultaneous use of organic additives, biodegradable vegetable oil, and recycling may lead to a synergistic effect with the reduced environmental pollution of cutting fluids. Jagadevan et al. [41] successfully treated waste metalworking fluid using a hybrid ozone–biological process. However, the study of Gerulova et al. [42] on the biodegradability of metalworking fluids was undertaken with a preliminary treatment stage of ozonation.

The objective of the present study is to assess the effectiveness of filtration and ozonation in improving the cleanliness and biological stability of metalworking fluids from an industrial band saw. An integrated approach to MWF decontamination that will extend its service life and reduce hygiene risks in industrial environments should be achieved.

## 2. Materials and Methods

This section presents the methodology of experimental research focused on characterizing the contamination of MWF and assessing the efficiency of purification methods, specifically filtration and ozonation.

### 2.1. Metalworking Fluid

**Figure 1:**
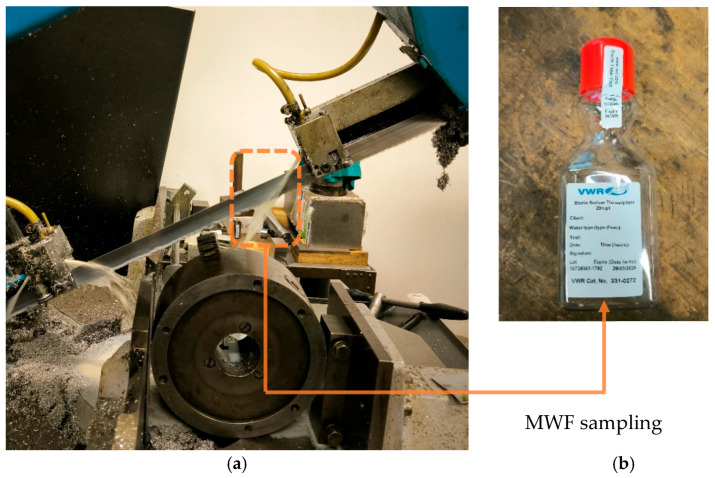
(**a**) Industrial band saw PILOUS ARG 300 Plus H.F. used in the experiment (PILOUS s.r.o., Brno, Czech Republic); (**b**) MWF sampling.

For the experimental study, a MWF was used as an integral technological component of the industrial band saw PILOUS ARG 300 Plus H.F. (PILOUS s.r.o., Brno, Czech Republic) (see Figure 1a), operating in a real production environment. The MWF used was EMULKANT UNI 500 N (FIPAS, s.r.o, Rokycany, Czech Republic), a water- and silicone-based cooling lubricant designed primarily for demanding cutting operations. Prior to use, the MWF was diluted with water to a concentration of 7%. According to the manufacturer, the MWF offers high stability, excellent lubricating properties, and effective corrosion protection.

During operation, a total of 12 MWF samples, each with a volume of 500 mL, were collected into sterile plastic bottles (see Figure 1b). Sampling was performed twice, with a one-week interval, to confirm the repeatability of the results. The MWF had been in use for 8 machine hours prior to sampling, ensuring natural exposure to biological and mechanical contaminants, including chips from both metal and composite materials.

The materials processed during the band saw cutting included primarily structural carbon steel (grade 11 523, equivalent to C45, Ferona, a.s., Prague, Czech Republic) and aluminum profiles (EN AW-6060, Ferona, a.s., Prague, Czech Republic). No copper-based materials were machined during the experimental period. We acknowledge that the presence of copper or its alloys could influence the microbiological profile due to its known biocidal properties. This will be considered in future studies evaluating MWF contamination of different groups of materials.

Determination of the type, size, shape, and chemical composition of microparticles. Various microscopic techniques were employed to characterize and analyze the micro particles present in the samples. A Stemi DV4 stereomicroscope (Carl Zeiss Microscopy GmbH, Oberkochen, Germany) was used for initial particle detection and to obtain a general overview of the samples. For more precise determination of particle quantity and size, a Keyence VK-X3000 confocal microscope (Keyence Corporation, Osaka, Japan) was utilized. Detailed analysis of particle type, size, shape, and chemical composition, as well as bacterial identification, was conducted using a Scanning Electron Microscope (SEM; Carl Zeiss Microscopy GmbH, Oberkochen, Germany) in combination with Energy-Dispersive X-ray Spectroscopy (EDS; Oxford Instruments NanoAnalysis, Concord, MA, USA).

### 2.2. Determination of Culturable Microorganisms

The procedure involved thorough homogenization of the samples using an IKA VORTEX GENIUS 3 shaker (IKA-Werke GmbH & Co. KG., Staufen im Breisgau, Germany) for 2 min. A dilution series was then prepared, from which 1 mL of the sample was aseptically transferred into a sterile Petri dish and overlaid with molten nutrient agar (standard medium without glucose; manufacturer: BIO-RAD, Hercules, CA, USA), tempered to 45 °C. The Petri dishes were incubated in a Memmert UF260 incubator (Memmert GmbH + Co. KG, Schwabach, Germany) at 22 °C for 68 h under aerobic conditions. After incubation, colonies were counted using a BioCote^®^ SC6+ electronic colony counter (Bibby Scientific Ltd, Staffordshire, United Kingdom) and results were expressed as colony-forming units per milliliter of sample (CFU/mL). The analysis also included the identification of specific bacterial strains based on cultivation characteristics and colony morphology.

**Figure 2:**
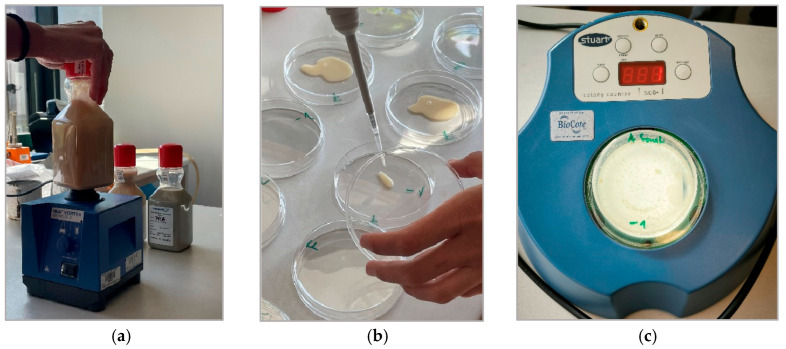
(**a**) Homogenization; (**b**) Sample application; (**c**) Counter.

The logical sequence of individual steps and the complete methodology for determining culturable microorganisms is illustrated in detail in Figure 2a–c. The procedure was carried out in accordance with the ČSN EN ISO 6222 standard [43]. After homogenization, the sample exhibited a pH of 8.9. Complete mixing was achieved after 2 min of vortex stirring, as confirmed by visual inspection and the homogeneous dispersion of particles throughout the sample.

### 2.3. Filter Materials Used

Six different types of commercially available nonwoven textile materials with nanofiber layers were selected for the experimental filtration of the MWF. These materials varied in their physical properties and in their declared intended use for filtration applications. The materials and their key properties are summarized in Table 1.

All tested materials consisted of a base layer made of spunbond polypropylene (PP) and a nanofiber layer made of polyamide 6 (PA6). The combination of these materials provides the desired mechanical strength, primarily contributed by the spunbond layer, along with filtration properties ensured by the chemically resistant nanofiber layer, which is resistant to the alkaline environment of MWF.

Each filter was disinfected before filtration. Subsequently, 500 mL of process liquid was poured onto each filter. Filtration efficiency was assessed using the gravimetric method. After filtration, the filters were dried, and the mass of captured impurities was measured using a precision balance (KERN ABJ 220–4NM, KERN & SOHN GmbH, Balingen, Germany). The filtration efficiency (η) was calculated as the ratio of the mass of impurities retained on the filter to the total mass of contaminants present in the unfiltered liquid.

The total solids content in the MWF prior to filtration was not determined using analytical methods such as drying residue or spectrometry. Rather, it was assessed by gravimetric comparison of the filter mass before and after filtration. This method allowed a comparative assessment of the filtration efficiency of different materials, rather than an absolute quantification of total solids.

### 2.4. Ozonation Method

To evaluate the effect of ozonation on the MWF, an ozone generator with a capacity of 1000 mg/h was used to ensure thorough mixing of ozone with the liquid. The ozonation process was carried out for 30 min. The pH of the fluid was monitored during and after treatment using pH indicator strips. After ozonation, samples of the fluid were collected and analyzed for the presence of cultivable microorganisms.

## 3. Results and Discussion

This study comprehensively examined the MWF used in the cutting of metal and composite materials, with emphasis on its physicochemical and microbiological characteristics, as well as the effectiveness of various filter materials and ozonation as treatment methods. The primary objective was to identify the main contaminants, specifically metal-derived micro particles and microorganisms, and to assess the performance of selected nonwoven fabrics in capturing these particles. In addition, the efficiency of ozonation in reducing microbial load was investigated. Filter materials were evaluated based on their ability to retain a broad range of particles of varying sizes and compositions, as well as on filtration time, fineness, and overall efficiency. Ozonation was examined as an alternative decontamination approach, and its effectiveness was subsequently assessed. The obtained results are presented and discussed in the context of relevant literature and existing expert sources.

Unlike milling, band sawing involves a continuous cutting action with lower cutting speeds and less aggressive chip formation. This may result in a higher proportion of elongated, fibrous particles and increased contamination from frictional delamination rather than from high-speed impact. Our findings confirm a predominance of acicular (needle-shaped) micro particles in the MWF, which supports this assumption. These morphological differences are attributable to the specific dynamics of the band sawing process.

It is well-documented that airborne particulate matter in enclosed environments, such as subway systems, contains high concentrations of iron oxides, predominantly generated by friction between rails and wheels. While some of the particles found in the MWF samples may originate from the surrounding environment through airborne deposition. The majority of the analyzed particles showed characteristics such as morphology and size, indicative of mechanical wear. Nonetheless, this observation reinforces the necessity of controlling both internal and external contamination sources in MWF systems.

While this study was conducted at a laboratory scale, both filtration and ozonation are scalable and can be adapted to real production environments. Filtration units using nanofiber membranes can be implemented in modular form directly within the MWF circulation loop. Ozonation systems are already available in industrial configurations and can be integrated with existing maintenance units. Energy demand is moderate, and no secondary chemical residues are produced, which supports industrial adoption. Future work will focus on pilot-scale implementation and cost-benefit analysis in continuous machining operations.

It is important to acknowledge that emerging net-shaping methods, including additive manufacturing (AM), can significantly reduce the necessity for subtractive processes such as machining. These technologies offer the advantage of near-net-shape part production with minimal waste and lower exposure to processing fluids. However, in many industrial sectors, especially when high dimensional accuracy and specific surface finishes are required, secondary machining remains essential. Therefore, optimizing MWF use and decontamination remains highly relevant until such time as AM can fully replace conventional processes.

### 3.1. Microparticle Analysis

Characterizing microparticles present in the MWF is essential for understanding their impact on machining quality and the service life of the fluid. These particles may come from tool wear, the machined material, or contamination from the surrounding environment. Detailed analyses were conducted using confocal microscopy and scanning electron microscopy (SEM) combined with energy-dispersive X-ray spectrometry (EDS), enabling precise characterization of particle shape, size, and chemical composition retained on the filter materials.

Morphological analysis of the microparticles revealed a broad size distribution within the process fluid. The smallest particle detected had a diameter of 7.3 ± 0.4 μm, while the largest measured particle reached 3530 ± 160 μm. The majority of particles were relatively small, with a predominance of those measuring less than 50 μm. Particle morphology varied with size, as summarized in Table 2.

Acicular particle morphology is indicative of abrasive wear and the presence of mechanical impurities. Additionally, particles exhibiting spiral and laminar geometries suggest material delamination caused by cutting or frictional forces. Large particles exceeding 1500 μm pose a significant risk of accelerated pump wear and filter clogging if not removed effectively.

**Figure 3:**
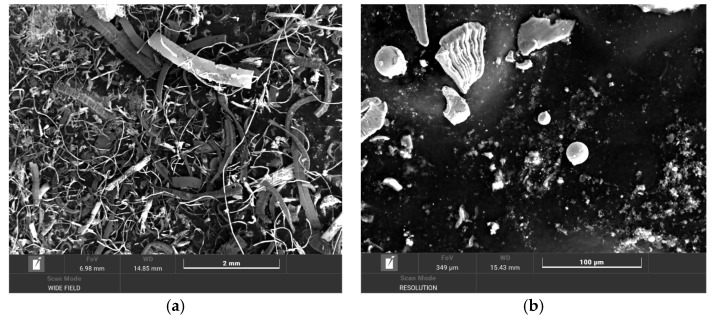
(**a**) Needle-shaped micro particles exhibiting both spiral (helical) and flat morphologies; (**b**) Round micro particles.

Morphological analysis of microparticles generated during the band saw-cutting process revealed considerable heterogeneity, with two predominant types. The first and most abundant type comprises needle-shaped microparticles (Figure 3a), which can be classified into helical and flat subgroups, reflecting the complex interactions between the cutting tool and the material as well as the dynamics of chip formation. The second major morphological type consists of round microparticles (Figure 3b), the presence of which suggests distinct abrasive wear mechanisms or specific conditions occurring during material separation.

The SEM images presented in Figure 3 were obtained using the secondary electron imaging (SEI) mode, which provides topographical contrast and was suitable for analyzing particle morphology and surface structure. This mode was selected to emphasize the shape and texture of the retained particles.

**Figure 4:**
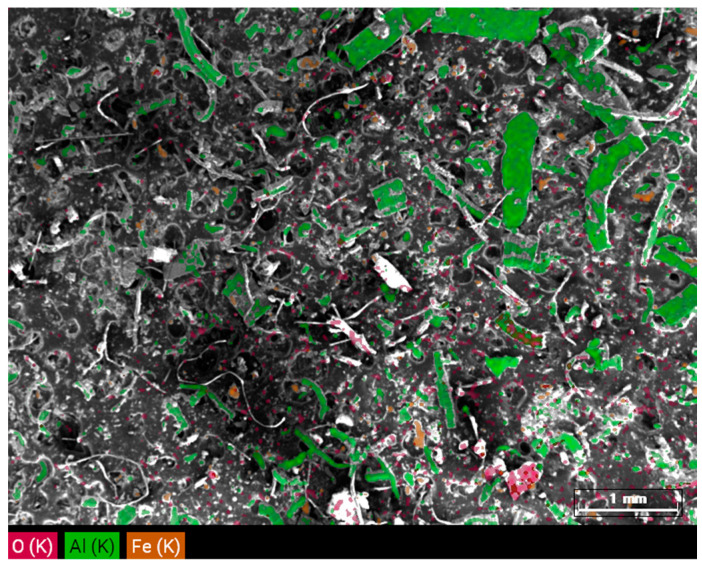
EDS analysis showing the mass percentages of chemical elements identified in the MWF micro particles.

Chemical analysis of microparticles using energy-dispersive X-ray spectrometry (EDS) revealed that particle composition varied across size fractions, with iron and aluminum identified as the primary elements in all categories. A detailed summary of the atomic and mass percentages of the detected elements is presented in Table 3. These results confirm that the particles originate from machined metal materials and wear of machine components. Oxygen was significantly represented, especially in particles smaller than 20 μm, frequently appearing as clustered formations. The presence of oxygen and aluminum suggests that aluminum oxides constitute a significant component of the trapped particles (Figure 4). Iron was detected in some samples and sulfur was observed in one case, particularly in fine particles.

Additionally, sulfur, calcium, and potassium were detected in low concentrations in the smallest particles (<20 μm). The presence of sulfur may indicate secondary aerosols or technological emissions. Carbon was also detected in some samples; however, this was attributed to the carbon adhesive tape used during sample preparation for SEM/EDS analysis rather than originating from the process fluid.

The identification and characterization of microparticles is a critical prerequisite for a comprehensive assessment of filtration efficiency. Particles smaller than 50 μm are of particular concern, as their large surface area enhances interactions with the microbial environment, potentially accelerating degradation processes within the fluid. The presence of oxidation and probable corrosion products such as oxygen and sulfur within the finest particle fractions may further compromise the chemical stability of the system. Effective control of solid particle contamination is essential for minimizing equipment wear and extending the service life of both the lubricant and the machinery [20,44]. Various treatment methods for removing impurities from waste MWF include adsorption, flotation, coagulation, membrane separation, and biological processes [11,21,45,46].

### 3.2. Filtration Analysis

Filtration is a key method for removing solid particles from MWF, helping to reduce equipment wear and extend the service life of both the fluid and associated machinery [47]. The experimental section of this study focused on a comprehensive evaluation of six different nonwoven filter materials. The determined parameters included filtration time, the quantity and size distribution of captured particles, the ability to retain fine particles, and overall filtration efficiency. Additionally, the composition of the retained contaminants was analyzed using SEM/EDS, as described previously.

Time-based permeability analysis of the filter materials revealed significant performance differences, as summarized in Table 4. The shortest filtration time (5 s) was recorded for the PEGATEX—PFNonwovens 408243 material. However, this filter primarily retained larger particles (over 250 μm) and failed to capture particles smaller than 20 μm, performing poorly in the removal of fine contaminants. In contrast, the Nano Medical 1055S09M063L000W1500 filter exhibited the longest filtration time (1 h 47 min, i.e., 6420 s) and appeared to retain the largest quantity of particles. The remaining materials demonstrated filtration times ranging from 22 to 2580 s.

Quantitative analysis of captured particles using confocal microscopy confirmed that most nanofiber-based materials—Nanovia Antivirus SMNF 57, Nano Medical 1080S03M014L000W1600, Nano Medical 1055S09M063L000W1500, Nano Medical 1080S03M014L000W1607, and Berry—were effective in capturing particles smaller than 20 μm. An exception was the PEGATEX—PFNonwovens 408243 material, which predominantly retained particles in the range 250–500 μm and failed to capture particles below 20 μm. Filtration fineness, evaluated based on the diameter of the five smallest particles captured, indicated that the Berry material achieved the highest resolution, capturing particles as small as 7.3 ± 0.4 μm, followed closely by Nano Medical 1055S09M063L000W1500 with 8.1 ± 0.9 μm. These smallest particles were most frequently retained at the point-bonded joints of the nonwoven fabrics, where the fiber structure is the densest.

Overall filtration efficiency, determined by the mass of impurities captured after filtering 500 mL of MWF, identified Berry filter as the most effective material, achieving 70.8% efficiency. Nano Medical 1055S09M063L000W1500 followed with 62.3%, while PEGATEX—PFNonwovens 408243 demonstrated the lowest efficiency at 38.5%. These results underscore the necessity of balancing filtration speed with effectiveness when designing optimized filtration systems. Filters with rapid throughput may not adequately capture fine particulate matter. Synthetic fibers are commonly used in industrial filtration applications due to their resistance to swelling and clogging when exposed to synthetic lubricants and specific contaminants. In contrast, cellulose-based materials may absorb liquid, swell, and reduce filtration efficiency. To enhance the overall quality of waste MWF, comprehensive regeneration systems often employ a combination of techniques such as mechanical filtration, membrane separation, and other advanced treatment methods [41,48]. A direct correlation between filtration time and pressure drop was confirmed. Filters with a higher density of nanofibers (e.g., Nano Medical 1055S09M063L000W1500) exhibited longer flow times (6420 s) and higher pressure drops (277 Pa), but captured a greater amount of fine particles. Conversely, materials with low pressure drop (e.g., Pegatex-PF) allowed faster fluid flow but had lower efficiency.

### 3.3. Microbial Analysis

Microbial contamination of MWF represents a significant challenge in the metalworking industry [13,17,20,43]. Due to their composition and water content, water-miscible MWFs create an ideal environment for microbial growth and metabolism. This can lead to biological degradation of the MWF and microbially influenced corrosion (MIC) of machine components.

**Figure 5:**
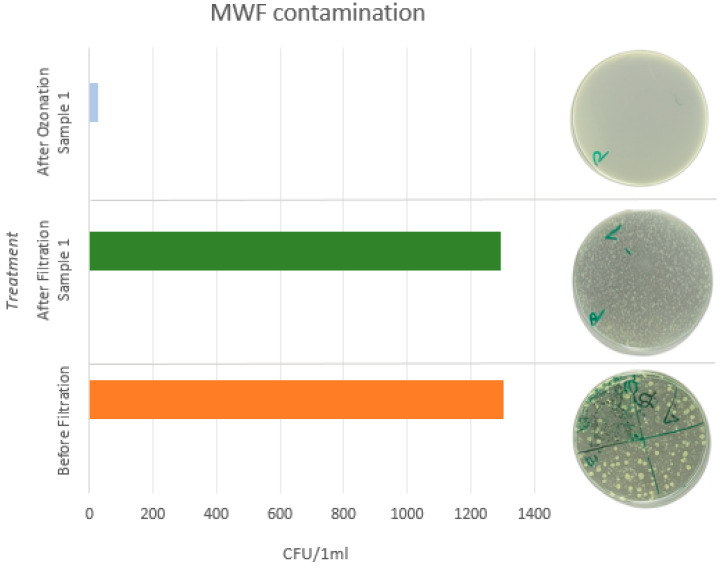
Graphical representation of MWF contamination after treatment, sample 1.

**Figure 6:**
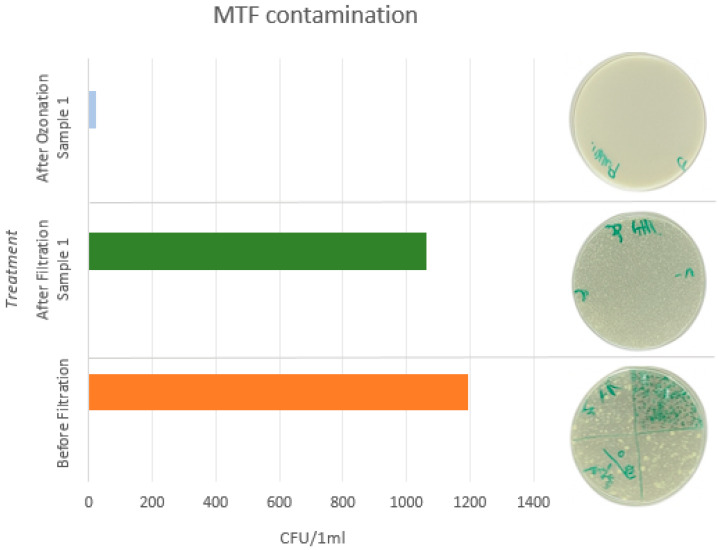
Graphical representation of MWF contamination after treatment, sample 2.

Microbiological analysis of the MWF samples prior to treatment revealed substantial microbial contamination [11,16]. The analysis was conducted in accordance with the ČSN EN ISO 6222 standard, which quantifies the number of culturable microorganisms. The results for two sampled MWFs are presented in Table 5 and Table 6, while Figure 5 and Figure 6 illustrate microbial growth following the second dilution.

In both the undiluted and first dilution samples, the culture plates were overgrown and exhibited turbidity after incubation, indicating high microbial activity. Quantification of colony forming units (CFU) was only feasible at the second dilution level (1:100), where CFU counts ranged from 1192 to 1304 CFU/mL. These values correspond to the high microbial loads commonly observed in contaminated MWFs, which are often reported to range from 10^4^ to 10^10^ CFU/mL.

A particularly important finding was the identification of *Pseudomonas aeruginosa* in the analyzed MWF samples. This species is frequently detected in MWFs and is well known for its resilience in harsh environments, as well as its ability to form biofilms on machine surfaces. Biofilms are structured, multicellular microbial communities embedded within a self-produced extracellular matrix. In the context of MWFs, biofilms often appear as slimy layers or flakes that can obstruct circulation systems, contaminate processed materials, and contribute to equipment degradation. Furthermore, they create favorable conditions for electrochemical corrosion of metal components [49,50].

Pseudomonas is the most commonly isolated genus in MWFs. It consists of Gram-negative, facultatively anaerobic bacteria that thrive in water-miscible fluids by metabolizing their organic constituents. Gram-negative bacteria in general are more prevalent than Gram-positive bacteria in such environments.

*Pseudomonas aeruginosa* is of particular concern not only due to its operational impacts but also due to its classification as an opportunistic human pathogen. It can cause infections in immunocompromised individuals, including wound and burn infections. Its presence in process fluids poses both hygienic and technical risks. Notably, P. aeruginosa is among the microbial contaminants implicated in the development of hypersensitivity pneumonitis, a serious respiratory condition observed in occupational settings with prolonged MWF exposure.

Traditional culture-based methods for assessing microbial burden, such as those employed in this study, have well-recognized limitations and frequently underestimate both the diversity and abundance of microorganisms present in MWFs. It is estimated that only a small proportion of microorganisms in such complex environments are culturable using standard laboratory media. As a result, reliance solely on culture-based techniques provides an incomplete picture of microbial contamination.

Advanced molecular techniques such as quantitative real-time PCR (qPCR) and fluorescence in situ hybridization (FISH) enable the detection and quantification of both culturable and non-culturable microorganisms, offering significantly improved accuracy and sensitivity. These methods are increasingly recommended for the routine monitoring of microbial contamination in industrial fluids.

For comprehensive microbial community profiling, metagenomic approaches—including 16S rRNA gene sequencing for bacterial communities and internal transcribed spacer (ITS) sequencing for fungal populations—provide deep insights into microbial diversity. These high-throughput sequencing techniques can identify a wide range of taxa, including many that are undetectable by traditional culturing methods, thereby supporting more effective risk assessment and fluid management strategies.

### 3.4. Ozonation Analysis

Ozonation represents a promising physical method for the decontamination of MWFs, and its application in this study demonstrated a substantial microbiological effect. A marked reduction in microbial load was observed after 30 min of ozone treatment, with colony-forming units (CFU) decreasing from initial concentrations of approximately 1300 CFU/mL to 25–29 CFU/mL, as presented in Table 5 and Table 6. This corresponds to a reduction in microbial load exceeding 95%. In contrast, filtration alone resulted in only a marginal decrease in CFU counts (e.g., from 1304 to 1296 CFU/mL), underscoring the superior efficacy of ozonation in microbial reduction.

Ozone is among the most powerful oxidizing agents and has demonstrated high effectiveness in the inactivation of a broad spectrum of microorganisms. It decomposes rapidly in aqueous solutions into molecular oxygen, leaving no harmful residues or secondary pollutants. The sterilizing potential of ozone has been validated in previous studies. For instance, complete microbial inactivation was achieved within 20 min of exposure in one study [27]. Other investigations report sterilization efficiencies reaching 99.99% after ozonation of MWFs. Although initial reductions in microbial counts may be minimal during the first two hours, prolonged ozonation (up to 24 h) has led to CFU reductions from 10^4^ to 10^2^ CFU/mL.

In addition to microbiological effects, the impact of ozonation on the physicochemical properties of the fluid was also monitored. Specifically, a pH decrease was recorded- from an initial value of 9 to 8 after 30 min of ozonation. This change is likely due to the partial neutralization of alkaline metabolic by-products resulting from microbial reduction processes.

From a practical standpoint, ozonation presents a technologically and economically viable method for the decontamination of MWFs [41]. Although the present study was conducted on a laboratory scale, the operational costs associated with ozonation were minimal, with the primary energy demand stemming from the ozone generator. One of the key advantages of this method is its ability to disinfect without leaving chemical residues, aligning well with current environmental and hygiene regulations. Ozonation demonstrates strong potential as a complementary treatment strategy aimed at prolonging the service life of MWFs, ensuring microbiological safety, and enhancing the overall operational stability of machining systems [38,41]. Furthermore, its combination with other physical methods, such as ultraviolet (UV) radiation, may lead to synergistic effects [6,21,51,52]. UV radiation induces damage to microbial DNA, and its efficacy in MWFs has been previously confirmed, particularly when combined with mechanical stirring to enhance exposure.

## 4. Conclusions

Morphological and chemical analyses showed that the majority of contaminants were particles smaller than 50 μm, primarily composed of aluminum and iron oxides. These particles come from wear of the machined materials and machine components.

Microbiological analysis confirmed a high level of contamination in the untreated MWF, with culturable microbial counts exceeding 1000 CFU/mL. The identification of *Pseudomonas aeruginosa*—a well-known opportunistic pathogen and a key contributor to bio deterioration in MWFs—underscores potential health risks for workers and emphasizes the need for robust microbiological control measures.

The highest filtration efficiency of about 70.8% *for particles ≥ 7.3 μm in diameter* was reached with the Berry filter, while the other five filters offered faster filtration times at the expense of reduced effectiveness in capturing fine particles. These results highlight the inherent trade-off between filtration speed and particle retention efficiency, which must be considered when selecting optimal filtration media for industrial applications.

Ozonation was found to be a highly effective method for microbial decontamination, achieving a reduction of more than 95% of culturable microorganisms. In contrast, filtration alone had only a minimal effect on microbial reduction, demonstrating the superiority of ozonation in decontamination.

These results support the implementation of combined maintenance methods for process fluids in an industrial environment. Moreover, reducing microbial and particulate contamination improves workplace hygiene and decreases health risks for workers.

The results of this study indicate that neither filtration nor ozonation alone is sufficient to maintain the long-term stability and quality of MWFs. An integrated maintenance approach is therefore recommended, combining efficient filtration, tailored to the specific particle types and size distributions, with complementary methods for microbial control, such as ozonation or UV radiation.

This study is one of the first to systematically evaluate the behavior and treatment of *MWFs* under real-life band saw operating conditions, combining multimodal contamination analysis with practical evaluation of filtration and ozonation. The proposed concept constitutes an innovative contribution to the field of fluid maintenance and workplace hygiene in machining.

## Figures and Tables

**Table 1:** Basic properties of the selected filter materials.

Filter Materials	Specification		Mechanical Properties	
			Area Density [g/m^2^]	Pressure Drop [Pa]
Pegatex-PFNonwovens 408243	PP spunbond	for filtration	17	5
Nanovia Antivirus SMNF 57	PP spunbond + PA6 nanofibers layers	for filtration	60	40
Nano Medical 1080S03M014L000W1600	PP spunbond + PA6 nanofibers layers	for filtration	30	10
Nano Medical 1055S09M063L000W1500	PP spunbond + PA6 nanofibers layers	for hepa	40	277
Nano Medical 1080S03M014L000W1607	PP spunbond + PA6 nanofibers layers	for filtration	30	17
Fiberweb, Synergex pure (Berry)	PP spunbond + PA6 nanofibers layers	for filtration	40	40

PFNonwovens a.s, Prague, Czech Republic; Nanovia s.r.o., Liberec, Czech Republic; Fiberweb, Berlin GmbH, Germany; Nano Medical s.r.o., Prague, Czech Republic.

**Table 2:** Size and description of microparticles.

Microparticle Size [μm]	Description of Microparticles
<20	Non-uniform particles with an indistinct, particulate form characteristic of dust.
20–50	Round-shaped particles constituted the majority of the sample.
50–100	The sample comprised a mixture of spherical and acicular particles.
100–250	Needle-like particles with characteristic acicular morphology were observed
250–500	Particles exhibiting spiral and planar morphologies were observed.
500–1000 and 1000–1500	Acicular morphologies predominated.

**Table 3:** Atomic and mass percentages of identified chemical elements in micro particles across different size fractions.

Chemical Element	Atomic Amount [%]	Mass Amount [%]
Oxygen (O)	37.14	23.95
Aluminum (Al)	56.26	61.19
Iron (Fe)	6.60	14.86

**Table 4:** Filtration time for different filter materials used in the treatment of MWF.

Filter Material	Filtration Time [s]
Pegatex-PFNonwovens 408243	5
Nanovia Antivirus SMNF 57	2580
Nano Medical 1080S03M014L000W1600	36
Nano Medical 1055S09M063L000W1500	6420
Nano Medical 1080S03M014L000W1607	22
Synergex pure (Berry)	1440

**Table 5:** Microbial contamination of MWF—Sample 1.

Dilution	Before Filtration Sample 1	After Filtration Sample 1	After Ozonation Sample 1
1 mL	Overgrown sample, emulsion turbidity	Overgrown sample, emulsion turbidity	Emulsion turbidity
1st dilution	Overgrown	Overgrown	63 CFU/mL
2nd dilution	1304 CFU/mL	1296 CFU/mL	29 CFU/mL

**Table 6:** Microbial contamination of MWF—Sample 2.

Dilution	Before Filtration Sample 1	After Filtration Sample 1	After Ozonation Sample 1
1 mL	Overgrown sample, emulsion turbidity	Overgrown sample, emulsion turbidity	Emulsion turbidity
1st dilution	Overgrown	Overgrown	51 CFU/mL
2nd dilution	1192 CFU/mL	1064 CFU/mL	25 CFU/mL

## Data Availability

The original contributions presented in this study are included in the article. Further inquiries can be directed to the corresponding authors.

## Abbreviations

The following abbreviations are used in this manuscript:

MWF	Metalworking fluid
